# Australian University Students’ Coping Strategies and Use of Pharmaceutical Stimulants as Cognitive Enhancers

**DOI:** 10.3389/fpsyg.2016.00277

**Published:** 2016-03-01

**Authors:** Charmaine Jensen, Cynthia Forlini, Brad Partridge, Wayne Hall

**Affiliations:** ^1^Centre for Youth Substance Abuse Research, Royal Brisbane and Women’s HospitalBrisbane, QLD, Australia; ^2^University of Queensland Centre for Clinical Research, Royal Brisbane and Women’s Hospital Campus, The University of QueenslandBrisbane, QLD, Australia

**Keywords:** prescription stimulants, cognitive enhancement, stress, coping, university students

## Abstract

**Background:** There are reports that some university students are using prescription stimulants for non-medical ‘pharmaceutical cognitive enhancement (PCE)’ to improve alertness, focus, memory, and mood in an attempt to manage the demands of study at university. Purported demand for PCEs in academic contexts have been based on incomplete understandings of student motivations, and often based on untested assumptions about the context within which stimulants are used. They may represent attempts to cope with biopsychosocial stressors in university life by offsetting students’ inadequate coping responses, which in turn may affect their cognitive performance. This study aimed to identify (a) what strategies students adopted to cope with the stress of university life and, (b) to assess whether students who have used stimulants for PCE exhibit particular stress or coping patterns.

**Methods:** We interviewed 38 university students (with and without PCE experience) about their experience of managing student life, specifically their: educational values; study habits; achievement; stress management; getting assistance; competing activities and demands; health habits; and cognitive enhancement practices. All interview transcripts were coded into themes and analyzed.

**Results:** Our thematic analysis revealed that, generally, self-rated coping ability decreased as students’ self-rated stress level increased. Students used emotion- and problem-focused coping for the most part and adjustment-focused coping to a lesser extent. Avoidance, an emotion-focused coping strategy, was the most common, followed by problem-focused coping strategies, the use of cognition on enhancing substances, and planning and monitoring of workload. PCE users predominantly used avoidant emotion-focused coping strategies until they no longer mitigated the distress of approaching deadlines resulting in the use of prescription stimulants as a substance-based problem-focused coping strategy.

**Conclusion:** Our study suggests that students who choose coping responses that do not moderate stress where possible, may cause themselves additional distress and avoid learning more effective coping responses. Helping students to understand stress and coping, and develop realistic stress appraisal techniques, may assist students in general to maintain manageable distress levels and functioning. Furthermore, assisting students who may be inclined to use prescription stimulants for cognitive enhancement may reduce possible drug-related harms.

## Introduction

### Background

The non-medical use of prescription stimulants by healthy individuals to enhance alertness, focus, memory, mood and other cognitive functions (Hildt and Franke, 2013) has been dubbed ‘pharmaceutical cognitive enhancement’ (PCE) by some bioethicists. The stimulants commonly used for PCE include those used to treat Attention Deficit Hyperactivity Disorder, such as Ritalin, Concerta or Adderall, and wakefulness promoting agents used to treat Narcolepsy, for example Modafinil (Repantis, 2013). There is some evidence that some university students are using these stimulants for PCE purposes (Bavarian et al., 2013; Mazanov et al., 2013; Singh et al., 2014; Ram et al., 2015). The assumption commonly made in ethical analyses of this practice is that PCE helps students to manage the performance demands of university life.

Although the prevalence of PCE is reported to vary widely between countries and institutions, it is often discussed as if it were very common in all academic and professional environments (Maier and Schaub, 2015). Australian studies have found low rates of (lifetime) PCE use in the general population at 2.4% (Partridge et al., 2012) and university students with an average of 1.4 to 4.4% for ‘study’ and/or ‘study only’ purposes (Mazanov et al., 2013). Discussions of prevalence and purported demand for PCE in academic contexts have been based on incomplete understandings of student motivations, and often based on untested assumptions about the context within which stimulants are used (Lucke et al., 2011; Zohny, 2015).

Many of these assumptions focus on the academic aspects of university life, such as improving grades, increasing or maintaining academic competitiveness, improving the ability to learn, and self-medicating for difficulties studying (Greely et al., 2008; Rabiner et al., 2009; Lucke et al., 2013). Much of the research focusing on student use of stimulants has focused on identifying the prevalence of prescription stimulant use, assuming that all stimulant use is motivated by the desire to enhance cognitive performance (Greely et al., 2008). Motives for PCE use may also incorporate recreational and lifestyle purposes (Partridge, 2013). In one of the only studies to report on the context of student prescription stimulant use, Hildt and Franke (2013) argue that “if one takes the users’ overall life situation into consideration, it seems that [they] perceive stimulants at least partly as beneficial for leading an ‘active life’ without being focused too much on academics” (Hildt et al., 2014). These findings call for a better understanding of the psychological and social factors that influence the use of prescription stimulants by university students for putative PCE purposes. Indeed, reasons for using stimulants as PCEs may encompass more than academic goals; they may also represent attempts to cope with biopsychosocial stressors in university life and to offset students’ inadequate coping responses, which, in turn, may affect their cognitive performance.

### Coping Strategies

**FIGURE 1:**
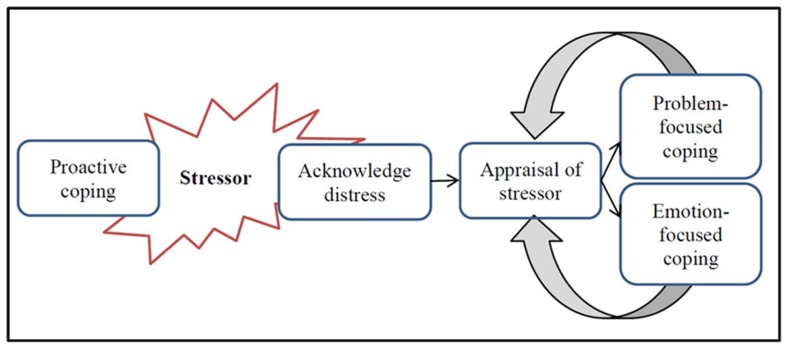
**Lazarus’ model of stress, appraisal, and coping**.

*Stressors* are situations or events that people perceive to be threatening to their physical, psychological, or social health, and may be acute or chronic. *Coping* refers to efforts to successfully navigate the challenges presented by stressors and alleviate associated distress (Snooks, 2009). Lazarus and Folkman identified that there are two common coping responses, *problem-focused* and *emotion-focused coping* (Lazarus and Folkman, 1984). Either or both of these coping responses represent a variety of coping strategies and can be applied in any given situation. The major difference between the two is whether or not the chosen coping response directly moderates the stressor. **Figure 1** briefly illustrates Lazarus and Folkman’s theory of choosing a coping response (Lazarus and Folkman, 1984).

*Proactive coping* refers to actions that prevent or minimize exposure to known stressors (e.g., developing good sleep, exercise, and nutritional habits) (Snooks, 2009). If a stressor cannot be prevented or minimized with proactive coping habits (e.g., sleep is interrupted by worry over deadlines leaving the person tired and prone to stress), it is appraised to determine what type of response is needed to minimize distress. There are two stages to appraisal; primary and secondary appraisal. Primary appraisal asks “what does this potential stressor mean to me?” which determines if the stressor is benign. If it is not benign, the secondary appraisal involves asking “what can I do?”

As new information about the stressor is acknowledged (e.g., realizing that an assessment will take more time to complete than first thought), reappraisal evaluates alternative responses and their perceived effectiveness to manage distress. Reappraisal is often cyclical as new information about the stressor comes to light, thus a review of the stressor and the evaluation of potential coping strategies is repeated (Snooks, 2009).

*Problem-focused coping* aims to directly manage a stressor to reduce distress. This response is effective when an individual has the ability to moderate the stressor (e.g., by starting an assignment earlier to reduce distress as the deadline nears). *Emotion-focused coping* aims to cope with the emotions and feelings aroused by the stressor. This response may be chosen when it is not possible to change or moderate the stressor itself (e.g., seeking social support when you are disappointed with an assessment grade that cannot be changed). Avoidance is a common emotion-focused response that allows temporary respite from the stressor, but at the cost of prolonging or amplifying distress (Snooks, 2009; Taylor, 2012). It is important to note that both coping responses can be helpful in particular situations and that there is not one coping strategy that is better at managing distress across all situations (Snooks, 2009).

Previous studies investigating stress and coping strategies of college students generally found that students’ perception of stress could predict their coping behavior (Kariv and Heiman, 2005), and that emotion-focused avoidant coping was more dominant than problem-focused coping. Some variables that mitigate the stress/coping relationship in previous studies are related to (1) gender, where masculinity is associated with problem-focused coping and femininity correlates with emotion-focused coping (Dyson and Renk, 2006; Brougham et al., 2009), although studies vary on this finding; (2) age, particularly 1st-year students (Kariv and Heiman, 2005; Dyson and Renk, 2006) where coping skills improve with practice and age (Snooks, 2009); and, (3) support, especially for those living away from family and friends during their studies (Dyson and Renk, 2006). Overall, improving coping behaviors, in particular problem-focused coping, appears to reduce depression symptomology (Steinhardt and Dolbier, 2008) and improve students’ academic grades (MacCann et al., 2011).

A lack of effective coping experiences and skills may mean that younger people are not as effective at choosing the most helpful coping responses to the demands of student life. Young people may also be more inclined to choose coping strategies, such as the use of PCEs to help manage distress where other coping strategies are overlooked or ineffective. The findings discussed in this paper are a part of a broader qualitative study investigating factors in the academic, psychological and social context that influence students’ interest in, and approaches to cognitive enhancement (including non-PCE methods).

### Study Aims

Here we explore our interview data to understand how students coping strategies may be related to PCE use, and how cognitive enhancement behaviors may be explained by current coping theories. We analyzed perspectives of university students on the demands of student life with the aims of identifying (a) what strategies students adopted to cope with the stress of university life, and (b) to assess whether students who have used PCEs exhibit particular stress or coping patterns.

## Materials and Methods

### Participants and Recruitment

Ethics approval for this study was obtained from the University of Queensland in accordance with the National Statement on Ethical Conduct in Human Research (Australia). We recruited students aged 18–24 that were actively enrolled in university courses. Participants were recruited for a research study about students’ study and health habits in several ways including: direct approach; posting flyers around campus noticeboards; the university’s online student blackboard; and, snowballing. A second round of purposive advertising was carried out to diversify the sample and recruit additional PCE users by modifying the advertisements to include “study drugs”. Approximately 250 students were screened in this second step to include more diversity in the use of caffeine, drugs, alcohol, and especially PCE use. Participants were compensated for their time with a $20 Coles Myer gift card. Recruitment ceased once there were no new themes emerging from the interviews and data saturation was achieved.

### Procedure and Materials

Participants were first asked to complete a 12-item demographic survey about: sex; age; residential postcode; Australian residency; ethnicity; caring responsibilities; years at university; current degree; host university; study-load; grade point average of previous semester; and, hours of paid employment per week. All interviews were digitally recorded to MP3 files and independently transcribed. We removed any identifying information and replaced the participant’s name with a number. Prior to commencement of the interview, participants were given an information sheet describing the study, what they would be required to do, and their rights as a participant. Interviews were conducted in 2013 by two members of the research team (CJ, CF).

The interview schedule focused on students’ experience and attitudes toward studying and how they manage the demands of student life. Questions were open-ended so that participants were able to provide more detailed responses and then prompted by the interviewer if more information was required. The semi-structured interview schedule consisted of several domains of interest: educational values (*“Why is it important for you to get an education?”*); study habits and achievement (*“Can you describe what your study habits look like?”* and *“What do you think makes a successful student?”*); stress management *(“How do you manage stress when you are studying?”*); getting assistance *(“Where would you get support if you needed it?”*); competing activities and responsibilities *(“Do you find yourself compromising other areas of your life to study, or do you compromise your study to do other things?”*); health habits *(“What do you sleep patterns look like?”*); and cognitive enhancement *(“Do you consume other things that help you stay alerts, concentrate or study?”*).

### Data Analysis

Interview transcripts were analyzed using NVivo qualitative data analysis Software (QSR International Pty Ltd. Version 10, 2012). Each transcript was read by two investigators (CJ, CF) to identify errors in transcription and remove identifying information. An inductive approach to thematic analysis was used to code student responses. The initial analysis identified patterns in how students coped, including the use of PCEs, which emerged as a coping response. A more focused analysis was carried out to identify different patterns of coping between PCE users and non-users. **Table 1** displays the coding structure for coping-related strategies. ‘Sources’ represent the number of participants who referenced the respective theme, and ‘references’ denote how many times the collective sample of participants referenced a theme, that is, one source may reference a particular theme or content multiple times.

**Table 1:** Thematic coding of coping strategies.

Coping theme	Sources	Reference
Emotion-focused coping	34	105
Avoidant Social support Switch tasks	32 12 3	83 18 4
Problem-focused coping	34	80
‘Cognition enhancing’ substances Planning, organizing and monitoring Exercise/sports/recreation Academic support	28 16 4 2	50 24 4 2
Adjustment-focused coping	8	10
Self-awareness/acceptance of limitations Perspective Spirituality	6 1 1	7 1 2

**FIGURE 2:**
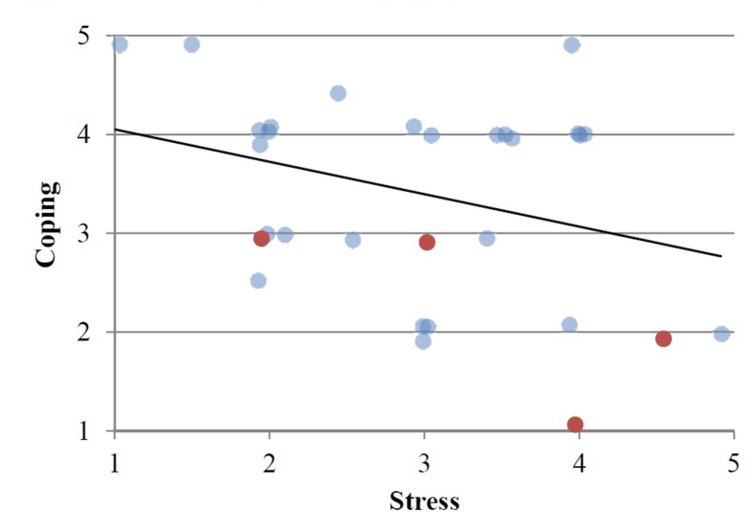
**Self-rated stress and coping levels.** Red markers indicate repeat PCE users. Bold line denote trendline.

During the interview, students were asked about their level of perceived stress and their perceived coping ability. A 5-point Likert scale (1 = lowest and 5 = highest) was used and then graphed in scatterplot form (refer **Figure 2**). Due to multiple data points overlapping and reducing the visual plotting of data points, scores were ‘jittered’ (Marinsek, 2015) by adding a random number between the range of -0.1 to 0.1 to visually indicate multiple data points.

## Results

We recruited 38 full-time university students with a mean age of 20.95 years (ranging from 18 to 24 years), with more females (*n* = 22) than males. The majority were Australian residents (*n* = 28), with the balance of international students originating mainly from America or Asia. **Table 2** displays demographic information about the students in this sample.

**Table 2:** Sample demographics.

Item	Males	Females	Combined average	Total
Sex	16	22	–	38
Mean years of age	20.8	21.1	21	–
International student	2	8	–	10
Average years at university	1	2.5	1.75	–
Average GPA	5.2	5.6	5.4	–
Employed weekly	11	17	–	28
Average self-rated stress	2.7 (0.98)	2.9 (1.1)	2.8 (1.0)	–
Average self-rated coping	3.7 (0.96)	3.4 (1.2)	3.5 (1.1)	–
PCE users	2	3	–	5

*Parentheses denote standard deviation of scores.*

The students interviewed displayed a negative relationship between their ability to cope with the demands of study and the level of stress that they reported experiencing. **Figure 2** displays students’ self-rated stress in relation to their self-rated coping levels. Students, who rated their ability to cope with stress as high, reported less stress than those who reported low coping ability. There were five students who had previous experience using PCEs, of which four were repeat users. The four repeat PCE users rated higher stress levels and lower coping ability than average.

We identified different ways that students reported coping with the pressure of study and managing stress to explain the correlation in **Figure 2**. Each student might use a combination of coping strategies but generally followed a dominant strategy. Through the thematic analysis of the interview transcripts, we identified two major coping themes, and one minor coping theme (see **Table 1**). These were, respectively: emotion-focused coping; problem-focused coping; and, adjustment-focused coping.

### Emotion-Focused Coping

Emotion-focused coping strategies changed how students felt in the *short-term* when they experienced stress. These included: avoidance; seeking support; and switching activities.

#### Avoidant Strategies

Avoidant strategies were the most common emotion-focused coping strategy. They involved the selection of activities that allowed students to avoid feelings of distress. The most common avoidant activities were: cleaning, eating, sleeping, checking/updating social media, and socializing.

Some students reported consistent use of avoidant behaviors. For example, *“I probably sleep more because I’m trying to avoid it* [study] *then, or just trying to take my mind off it because it’s the only time you don’t really think about it”* (11). Others avoided the task at hand with, “*Anything so cleaning the house, surfing the internet, watching movies, going for a walk, cleaning the car, anything”* (03).

Avoidant strategies provided short-term relief by allowing the student to avoid the unpleasant feelings associated with the study task. However, students did not necessarily enjoy the avoidant task, such as Student 27 who commented that: *“It’s like – the horrible thing is, I might normally enjoy watching YouTube videos or whatnot, but because I’ve got stress in the back of my mind, all enjoyment is kind of sucked out of it and it just becomes an avoidant activity*”, but this often does not reduce the stress afterward: *“I could be playing browser based games, up to three in the morning. Just could be, just to forget about* [what I have to do]*, yeah, and that obviously stresses me out”.*

Students often described avoidant strategies as unplanned. The duration of avoidant activities was often uncontrolled, potentially costing the student more time than anticipated. One student explained that: *“Distractions probably go through the roof, just procrastination. Finding other things to do during the break but then the break seems to get longer and longer just because you’re really sick of studying all day. … It’s good to have the relaxation period but then because it goes on for so long you’re pressured even more to study even harder and longer”* (03).

Many students who used these strategies also reported that the stress of an approaching deadline motivated them to start study tasks, and that they probably need the distress to get started: *“I’d just rather go out with my friends and do stuff. I knew it was bad and it stresses me out but I still did it. … Yeah I work better under pressure I think”* (15).

The students who used PCEs predominantly used avoidant strategies and experienced additional stress when little progress had been made toward meeting a looming deadline. When this strategy no longer worked, Student 29 notes that, *“I usually take it* [Concerta] *like before that* [stress] *happens just so I can start studying. But usually it just gets too close* [to the deadline], *even though I have that and I can study for hours it’s still really stressful.”*

#### Social Support

Seeking social support was another emotion-focused coping strategy. Students sought support from friends, family, and peers about how they felt. For example, *“Yeah if I’m having panic attacks and stuff mum will help just calm me down and put things in perspective, because it’s just because I get overwhelmed and worried I won’t get everything done… Yeah or someone to just talk at so I can put all my things in order and then realize I’m okay”* (15).

Seeking support often had a positive outcome because it allayed some of the unpleasant feelings and provided new ways of seeing or approaching the task. Student 35 noted that, “*I guess my family’s pretty, like they’re all pretty nice, and comforting so just being at home is quite good.”* Other types of social support allowed students to get back to the task in a more positive way, for example: “*So I think talking helps … So I was really feeling bad afterward because he had to cope with me. But still, afterward you feel better because you talked with somebody about your day and what you still have to do. So that helped me, that somebody is there and says, well yeah, can I help you with that?”* (08).

#### Switching Activities

Students often described switching activities if their study task was creating stress. This strategy uses a planned or controlled break away from the task to undertake activities that were not designed to progress the task directly but allowed the student to return to the task feeling less stressed. For example: *“I find music really helps with stress, especially if it’s – if there’s not much I could really do in that situation, like if I just need to alleviate that anxiety I would just listen to music that I like for maybe 10 min and then I get better. Sometimes talking to a friend could work too or just – sometimes when it’s really – sometimes you think you don’t have time to do anything but then your brain just can’t work. I find that during those times it’s better to just remove yourself and say okay like take a break, take a walk somewhere and then come back to it”* (06).

Switching activities differed from avoidant strategies because the intention was to relieve mental weariness rather than simply to avoid task-related stress. It was also a short-term strategy that often allowed the student to return to dealing with the stressor sooner. It was therefore more effective in addressing the task than other emotion-focused coping styles that did not moderate the stressor.

#### Problem-Focused Coping

Problem-focused coping strategies moderate the stressor directly, resulting in better *long-term* management of the stressor. Students described many ways in which they directly managed the stressors in their university lives.

#### Substance Use

Students were specifically asked if they used particular substances of any kind to help them study. The use of substances with the specific purpose as a study aid was the most commonly referenced problem-focused coping strategy across the sample. **Table 3** lists the substances that students were using to enhance their study performance during the academic semester the interview was conducted. This list does not reflect prior broader experience with substances for PCE or recreational purposes, which is represented in **Table 1**.

**Table 3:** Substances currently used by university students as a problem-focused coping strategy.

Substance	Sources	Reference
Coffee	11	12
Energy drinks	7	12
Food (chocolate, fruit, juice)	5	6
Tea (black and herbal)	5	6
Caffeine pills	4	5
Prescription stimulants	2	2
Essential oil	1	1
Fish oil supplement	1	1
**Total**	**28**	**45**

These putatively *cognition-enhancing* substances can be seen as a problem-focused coping strategy that allows students to moderate their distress by directly working on study goals. For instance, ‘tiredness’ was a common obstacle to working on academic tasks. Students reported using substances *“… if I’m really tired and I have to do something”* (11) or *“if I have an assignment due the next day, I’ll buy an energy drink just to stay awake the whole night and make sure I don’t fall asleep”* (21).

Students were aware of potential cognitive enhancing properties of the substances they used and made a distinction between their use as a study aid and use for other purposes, such as enjoyment or long-term or general health benefits. For example, one participant acknowledged that they used energy drinks “*To help pick me up, not for the taste. I know they’re terrible for you, but sometimes you’ve got to do it, I find anyway*” (34).

Some participants’ use of cognitive enhancing substances and dietary supplements, although known for various long-term health benefits, were used specifically for cognition enhancing purposes: “*I just take fish oil – just because of the – I know it’s quoting from the neuroethics lecture about drugs that enhance cognition”* (22).

Use of multiple substances was reported by some participants, especially those who were inclined to use prescription stimulants as PCEs or other illicit substances, as Student 24 illustrated: *“So I was using energy drinks for the most part. I managed to for the final semester of my honors I did manage to get a hold of some dexamphetamines – so some ADD medication. …Then at the very end I was also using No Doz* [caffeine pills] *as well.”*

#### Planning, Organizing, Monitoring and Reward

Planning, organizing, monitoring and reward were strategies that students used to moderate the stress of study. This allowed students to forecast potential stressors, plan around them and monitor their progress and associated stress. A common theme in planning was being aware of the time required for tasks: *“So if I have a harder assignment I’ll start it earlier because I know that it’s going to take me longer to figure it out and I give myself more time to stop working for the day and start tomorrow if I get too stressed”* (02).

Organization around tasks improved students’ execution by planning when something needed to be done and how long it would take. It also identified the step-by-step process required to complete the task, therefore minimizing distress: *“I plan a lot. So I know – I had a big plan the weeks before exam block, and even during exam block, when I would have to study for the next thing. So I knew when I had to do what. So that way I wasn’t like getting to the library and going, what do I have to do now?”* (34).

Monitoring performance and rewarding achievements were another theme that reinforced planning and organizing tasks. One student commented on having a very visual method for monitoring and appraisal of stress and progress: *“Like often I’ll leave a pile of things on my desk of what needs to be done when I start studying next and if that pile starts building up I feel stressed about it but if I’m on top of it and I’ve been doing like my 3 h a day, you know, or something like that I’ll feel good about it and I’ll be able to take a break and pull back from it and it will make me feel okay about it”* (25).

Reward for achieving task-related goals appeared to be associated with being able to withstand distress created by the task. It also enabled the students to fully enjoy the pleasures deferred without the residual distress of incomplete tasks often mentioned by emotion-focused-avoidant-type students. For example, Student 11 stated that *“A lot of the times I’ll say if I get this much done, then I can go to whoever’s party on the weekend. Obviously, if I had heaps of work to do, then I can only go for a bit, or I can’t go.”*

#### Exercise

Exercise was a minor theme mentioned as a tool to improve cognitive performance. It can be considered a problem-focused coping strategy that improves general and brain health, associated with cognitive improvements, for example, *“I think it just gets the blood flowing and just gets my brain working again, like if I’ve been just sort of watching videos on the Internet or I’ve just been on Facebook and my brain’s just like* [makes flat sound]*, going for a run really helps kick it back into gear I think”* (33).

#### Academic Support

Seeking academic support for study-related tasks helped students minimize and alleviate the stress in carrying out the task. This kind of support may not directly soothe or avoid unpleasant feelings about a stressful task, but mitigated the distress associated with the task, which in turn moderated the stressor: *“So if we have an issue we go to* [the college advisor] *and a couple of weeks ago I was just like I’m falling apart, I’m going to rip all my hair out, not good stress management, so she was like look, it’s just a weekly thing, if you hand it in a couple of days late that’s fine, just get it in. So it wasn’t a big deal, she still passed me”* (07).

Pharmaceutical cognitive enhancement users in this sample were aware of the various resources available for support but were more hesitant to seek support from services, peers, or teaching staff. These students noted that either they didn’t need extra support or didn’t think that they should need extra support: *“I know that I’m a bit smarter than anyone else… I should be able to think through my own problems. Yeah, I just feel like I should be able to figure it out myself”* [32], or felt uncomfortable about seeking it, *“I would literally just try to figure it out myself, like I just don’t like discussing my study habits or like learning with other people”* (29).

### Adjustment-Focused Coping

Adjustment-focused coping was a minor theme. This coping strategy changes the way one thinks about stressors. This coping style takes a more rational/cognitive perspective than the behavioral emotional- or problem-focused coping. Adjustment-focused coping was the least common strategy used by students but some shared lessons on how their perspective on stress had changed during their university studies. For example, one student learned that: *“From that point on I said, it’s not worth stressing yourself out because it’s not really going to change the quality of your work regardless. So it was very much a conscious thing. So now I don’t stress anymore”* (24). Another student spoke about how spirituality helped them to manage stress: *“I try my best to get my spiritual life up with it. I just believe in the degree. I can only plan and do my best, but the final decision is not up to me”* (12).

## Discussion

The students in our sample used a range of strategies to manage the stressors in student life. As per Lazarus’ model of stress, coping, and appraisal, the majority of coping strategies were aligned with problem- or emotion-focused coping styles (Lazarus and Folkman, 1984). Strategies within these coping styles are similar to those validated in other studies. The most common coping style used by the students in our sample was emotion-focused coping, particularly avoidant coping. This strategy is better suited to dealing with stressors that are not able to be directly moderated. Therefore, it focuses on managing the unpleasant feelings in the short-term caused by the stressor.

Problem-focused coping was also common and was predominantly substance-based or involved planning and monitoring strategies. This form of coping focuses on changing the stressor directly thereby reducing some of the distress associated with the completing the task. This strategy is generally better suited to stressors that can be directly moderated, therefore managing stress in the long-term by reducing the challenge presented by the stressor.

Adjustment-focused coping was a minor theme in our data with self-awareness and acceptance of limitations being the most common strategy in this theme. This strategy does not directly seek to relieve unpleasant emotions or moderate the stressor itself. It changes the perception of task-related distress without behavioral action, as emotion- and problem-focused strategies do. A majority of studies on coping have looked at emotion-focused coping or problem-focused coping and few studies review a third coping strategy such as this. It is unclear if this type of coping is a subset of the two dominant strategies, or rather an aspect of the appraisal function as in Lazarus’ model of stress, coping and appraisal.

We observed that students often used more than one style of coping for a stressor but exhibited a dominant strategy of coping. The dominant strategy was used until the stressor dissolved or until the strategy no longer minimized the distress, at which point reappraisal suggests that a new strategy needed to be employed. This process generally resulted in the use of more problem-focused strategies as the student context requires that some tasks had to be tackled (i.e., successfully navigating the challenges of the stressor). We found that the coping strategies of the regular PCE users in this sample were dominated by emotion-focused coping. Avoidant coping strategies were used until they no longer minimized distress at which point an alternative approach was chosen, such as using PCEs.

Understanding the behavioral cycle of anxiety may help explain the avoidant coping strategies students frequently adopt to manage the demands of study. Allport noticed a cycle of distress in which avoiding distress became a self-maintaining behaviour independent of the stressor (Allport, 1937; Seif and Winston, 2014). Students using emotion-focused avoidant coping responses often found some short-term relief from stress by focusing on the feelings aroused by the stressor. This, in turn, encouraged them to use avoidance coping strategies in the future.

However, avoidant coping does not diminish the original stressor so the cycle repeats as feelings of distress resurface (i.e., assessments still need to be completed). While this coping response is maintained, students miss opportunities to develop other strategies that may reduce distress more effectively. Students who are coping in more problem-focused ways use longer-term coping responses and dimish distress, subsequently reducing the original stressor’s challenge and reinforcing problem-focused coping strategies.

Using PCEs may be a way to directly moderate the stressor and facilitate work on their task. Although this may be perceived as an effective way to directly moderate the stressor (that is, a problem-focused coping strategy), it may not be a healthy long-term strategy. The increased prescribing of ADHD medications over the last decade in Australia has opened up the potential for diversion or normalization of prescription stimulants use in society (Kaye and Darke, 2012). Kaye and Darke (2012) report that many users obtain prescription stimulants from friends with prescriptions. Whilst there is a lack of consensus about the efficacy of prescription stimulants to enhance cognition in healthy individuals, there is better evidence for their adverse side-effects and abuse potential (McCabe and Teter, 2007; Weyandt et al., 2013).

To our knowledge, previous studies have not used health-psychology to understand PCE practices. Further studies investigating the relationship between coping and PCE use would be informative. Specifically, further investigation is required to understand if adjustment-focused coping is a subset of appraisal, such as to what extent do students perceive that they have control over potential coping responses and the capacity to moderate the stressor (i.e., “what can I do?”).

### Limitations

Given that there is some overlap between emotion- and problem-focused coping and both can alleviate distress and moderate the stressor our categorisation of PCE as problem-focused coping would benefit from further confirmation. For example, one student may use PCEs to work directly on an assignment but PCEs may also improve mood, which in turn makes it easier to work on a task (Vrecko, 2013). In this study, we defined coping styles based on the responses to stressors rather than the outcome of the response. This is in line with the behavior-oriented coping strategies in Lazarus’ model of stress, appraisal, and coping, which does not seek to identify a correct coping response. Instead, it attempts to find the most effective coping response to the stressor in its situational context.

There are some methodological aspects of this study that limit the generalizability of the results. There was a time limit on interviews of 1 hour. In order to collect a breadth of data, some topics were not discussed in depth. It is also unclear how broadly the results apply to the larger population of university students.

This study included a small number of PCE users. We attempted to increase the number by screening approximately 250 students to find more PCE users, however, our findings our findings are consistent with the only two other Australian studies that have found low rates of PCE prevalence in the general population (Partridge et al., 2012) and university students (Mazanov et al., 2013).

## Conclusion

We interviewed 38 university students, five of whom had experience using PCEs, about how they managed the demands of student life. We found that students who rated higher stress generally rated lower coping ability. The students in this sample used a range of strategies to manage the stress of student life. Both emotion- and problem-focused coping were styles students used to manage stress in their everyday life, often preferring one over the other until it no was longer effective at minimizing stress.

Pharmaceutical cognitive enhancement users reported higher levels of stress and lower levels of ability to cope than the sample average. They preferred to use avoidant emotion-focused coping strategies until they were close to deadlines where they then used stimulants as a problem-focused alternative coping strategy to moderate their stress. This may expose PCE users to additional health harms that may arise from the regular use of prescription stimulants as a coping strategy.

Our study suggests that students who choose coping responses that do not moderate the stressor, where possible, may cause themselves additional distress and avoid learning more effective coping responses. Helping students to understand stress and coping, and develop realistic stress appraisal techniques, may assist students in maintaining manageable distress levels and functioning both in and out of the university environment. Furthermore, assisting students who may be inclined to use prescription stimulants for cognitive enhancement may reduce possible drug-related harms.

## Author Contributions

CJ came up with the conceptual framework for the paper based on findings in a broader study. WH reviewed a draft abstract and approved the research idea. CJ carried out majority of data collection, analysis, and drafting of manuscript, with CF taking a smaller role in collecting data, analyzing data and writing the manuscript. BP helped CJ early on articulate the concept and provided significant early critique that shaped the paper. WH has provided expert knowledge, guidance, and revisions to the paper.

## Conflict of Interest Statement

The authors declare that the research was conducted in the absence of any commercial or financial relationships that could be construed as a potential conflict of interest.
